# The effects of lower limb blood flow restriction training on post-activation potentiation enhancement and fatigue: a meta-analysis

**DOI:** 10.3389/fphys.2026.1834109

**Published:** 2026-07-08

**Authors:** Xinrui Miao, Jiajun Hou, Wenqing Chang

**Affiliations:** 1 Department of Physical Education, Kunsan National University, Gunsan, Republic of Korea; 2 College of Physical Education, Putian University, Putian, China

**Keywords:** blood flow restriction training, meta-analysis, post-activation potentiation, rest interval, squat

## Abstract

**Objective:**

This study aims to systematically explore the effects of lower limb blood flow restriction (BFR) training on post-activation potentiation enhancement (PAPE) and the Rating of Perceived Exertion (RPE) through meta-analysis, and to identify optimal BFR protocol parameters, so as to provide scientific evidence for strength and conditioning training and rehabilitation interventions.

**Methods:**

A systematic search was conducted in PubMed, Web of Science, Scopus, and EBSCO databases to retrieve randomized controlled trials (RCTs) investigating the acute effects of lower limb BFR training on PAPE and RPE. The search timeframe covered the inception of each database to Dec 1, 2025. The Cochrane Collaboration’s Risk of Bias Tool was used for literature quality assessment. Review Manager 5.4 and Stata 17.0 software were employed for statistical analysis, including heterogeneity testing, subgroup analysis, sensitivity analysis, and publication bias assessment.

**Results:**

A total of 15 eligible RCTs were included, involving 244 subjects aged 17–35 years. Meta-analysis results showed that lower limb BFR training significantly enhanced PAPE-related explosive performance [*SMD* = 0.55, 95%*CI* (0.08, 1.01), *P* = 0.02] with high heterogeneity (*I*² = 78%, P < 0.001). Subgroup analysis revealed that the optimal parameters for PAPE enhancement were: multi-joint movements such as squats [*SMD* = 1.20, *95%CI* (0.78, 1.63), *P < 0.001*], rest intervals > 4 min [*SMD* = 0.98, *95%CI* (0.67, 1.30), *P < 0.001*], occlusion pressure of 40–60% arterial occlusion pressure (AOP) [*SMD* = 0.83, *95%CI* (0.29, 1.36), *P* = 0.002]. Countermovement jump (CMJ) was the most sensitive outcome measure for PAPE assessment [*SMD* = 0.83, *95%CI* (0.44, 1.21), *P <* 0.001]. Notably, several subgroups were based on only 1–2 primary studies, and these findings should be interpreted with extreme caution due to limited sample size and evidence volume. Regarding RPE, BFR training significantly increased subjective fatigue [*SMD* = 0.82, *95%CI* (0.39, 1.26), *P* < 0.001] with moderate heterogeneity (*I*² = 60%, *P* = 0.001). Subgroup analysis indicated that more pronounced fatigue perception was observed in knee flexion/compound exercises [*SMD* = 0.91/1.54, P <0.001], rest intervals ≤ 4 min [*SMD* = 0.79/1.16, *P* = 0.05], low exercise intensity (< 40% 1RM) [*SMD* = 0.86, *95%CI* (0.30, 1.42), *P* = 0.003], and occlusion pressure > 60% AOP [*SMD* = 1.37, *95%CI* (0.80, 1.94), *P <* 0.001]. Sensitivity analysis confirmed the stability of the results, and funnel plots combined with Egger’s test showed no significant publication bias (*P* > 0.05).

**Conclusion:**

Lower limb BFR training can effectively induce PAPE, with optimal effects achieved through multi-joint movements, rest intervals > 4 min, 40–60% AOP, and 40–70% 1RM intensity. Meanwhile, BFR training significantly elevates subjective fatigue (RPE), which is more prominent under conditions of knee flexion/compound exercises, shorter rest intervals, low intensity, and high occlusion pressure (> 60% AOP).

**Systematic Review Registration:**

https://inplasy.com/inplasy-2026-5-0041/, identifier INPLASY202430008.

## 1 Introduction

Post-activation potentiation (PAP) is a well-characterized physiological phenomenon whereby acute high-intensity or submaximal conditioning contractions elicit a transient enhancement in subsequent muscular explosive performance, a mechanism central to optimizing warm-up protocols and athletic performance in sports requiring rapid force and power production (Blazevich et al., 2019). Notably, this study strictly distinguishes PAP from post-activation performance enhancement (PAPE) according to the authoritative definitional framework: PAPE refers to the underlying neuromuscular potentiation mechanism, whereas PAPE represents the observable, externally measured explosive performance improvements (e.g., CMJ and VJ outcomes) induced by PAPE (Seitz et al., 2016). All jump-based outcomes analyzed in the present study reflect PAPE rather than the intrinsic PAPE mechanism. As a key modulator of neuromuscular function, PAPE has become a focal point in exercise physiology and strength and conditioning research, with its magnitude and duration shaped by a cascade of factors including conditioning load, rest interval, and individual training status (Wilson et al., 2013; Cuenca-Fernández et al., 2017). Concurrently, subjective fatigue—an integrative perceptual response to physical exertion encompassing neuromuscular, metabolic, and psychological components—acts as a critical counterbalance to PAPE efficacy: excessive fatigue induced by conditioning contractions can mask potentiation effects, creating a dynamic trade-off that dictates the practical utility of PAPE-inducing interventions in athletic and clinical settings (Tillin et al., 2009; Prieske et al., 2020).

Blood flow restriction (BFR) training, a novel exercise modality involving the application of external pressure to proximal limbs to limit arterial inflow and venous return during low-load resistance exercise, has emerged as a transformative approach to elicit physiological adaptations traditionally associated with high-load training while minimizing mechanical stress and injury risk (Loenneke et al., 2015; Patterson et al., 2019). In recent years, research has extended the application of lower limb BFR training beyond chronic muscle hypertrophy and strength gains to its acute effects on neuromuscular potentiation, with preliminary studies suggesting that BFR may modulate PAPE responses by augmenting metabolic stress, muscle fiber activation, and neural drive—pathways distinct from those of conventional high-load conditioning (Lesinski et al., 2013; Wang et al., 2023). Unlike high-load resistance training, which often induces significant acute fatigue that blunts PAPE in untrained or recreationally active populations, lower limb BFR training at low relative loads offers a potential strategy to optimize the potentiation-fatigue balance, making it a promising intervention for athletes, rehabilitation patients, and individuals with limited load-bearing capacity (Vechin et al., 2015; Zheng et al., 2025).

Despite the growing interest in the intersection of BFR training and PAPE, the extant literature on lower limb BFR training’s effects on PAPE and subjective fatigue remains fragmented and inconsistent. Individual studies have reported divergent findings: some demonstrate enhanced PAPE magnitude and prolonged potentiation windows with BFR training, accompanied by moderate subjective fatigue (Wilk et al., 2022; Cornejo-Daza et al., 2025), while others show no significant potentiation effects or even elevated perceptual fatigue compared to conventional non-BFR conditioning (Cook et al., 2014; Nancekievill et al., 2025). These discrepancies are attributed to substantial variability in study design, including BFR pressure parameters (absolute vs. relative arterial occlusion pressure), conditioning exercise protocols (squats, leg presses, dynamic vs. isometric contractions), rest intervals post-conditioning, and participant characteristics (training status, age, sport-specific background) (Dong et al., 2024; Liu et al., 2024). Additionally, most existing investigations are small-sample, single-center studies, and few have systematically quantified the relationship between BFR-induced PAPE changes and subjective fatigue perceptions—a critical gap given the practical need to balance physiological potentiation with perceptual tolerance in real-world training and rehabilitation.

To date, no comprehensive meta-analysis has specifically focused on the dual effects of lower limb BFR training on PAPE and subjective fatigue, nor has there been a systematic exploration of moderating factors that may shape these outcomes. Addressing this gap is essential to resolve inconsistencies in the current evidence base, establish evidence-based guidelines for BFR training protocol design to optimize PAPE, and clarify the perceptual fatigue costs associated with BFR-induced potentiation. The present meta-analysis therefore aims to systematically synthesize the available empirical evidence to determine the overall effects of lower limb BFR training on PAPE-related neuromuscular performance outcomes and subjective fatigue. Secondary objectives include investigating the moderating roles of key study design and participant characteristics (e.g., BFR pressure, conditioning load, rest interval, training status) on these effects, and identifying gaps in the current literature to guide future research. By quantifying the potentiation-fatigue response to lower limb BFR training, this study seeks to provide a rigorous evidence base for strength and conditioning specialists, exercise physiologists, and rehabilitation clinicians to inform the design of effective, fatigue-mitigated PAPE-inducing interventions.

## 2 Materials and methods

### 2.1 Search strategy

A systematic literature search was conducted across four electronic databases (Web of Science, PubMed, CNKI and EBSCO) in accordance with the Preferred Reporting Items for Systematic Reviews and Meta-Analyses (PRISMA) guidelines (Moher et al., 2009). This systematic review was prospectively registered in 2024 (INPLASY202430008), and the final literature retrieval was conducted on December 1, 2025, generating an initial total of 1563 records.

The search strategy was rigorously designed based on the PICO (Population, Intervention, Comparison, Outcome) (Li et al., 2014) framework, tailored specifically to the core objectives of this meta-analysis:

Population: Healthy humans;
Intervention: Lower limb blood flow restriction (BFR) training as a post-activation potentiation (PAPE) induction strategy;
Comparison: Conventional high-load resistance training, low-load resistance training without BFR, or no intervention;
Outcome: PAPE-related neuromuscular performance and Rating of Perceived Exertion (RPE) for fatigue assessment.

To ensure comprehensiveness and consistency across databases, the optimized English search terms were structured as follows: (“Blood flow restriction” OR “BFR” OR “Occlusion training” OR “Kaatsu training”)AND (“Post-activation potentiation” OR “PAPE” OR “Post-activation performance enhancement” OR “PAPE”)AND (“Lower limb” OR “Leg” OR “Knee” OR “Squat”)AND (“Rating of Perceived Exertion” OR “RPE” OR “Perceived exertion”)AND (“Randomized controlled trial” OR “RCT” OR “Controlled trial”).

Methodological note on search limitations: The current Boolean search combined RCT methodological filters and RPE outcome terms, which may reduce search sensitivity. RCT-specific keywords could exclude eligible randomized trials indexed without these exact labels; meanwhile, RPE-related terms may omit studies that only reported PAPE outcomes but did not assess perceived exertion. Due to the heterogeneity of literature indexing rules across databases, we retained the original search syntax. To mitigate omission bias, we additionally performed a supplementary manual screening of reference lists from included reviews and primary studies, and cross-checked grey literature. No formal sensitivity analysis for search strategy was conducted in this study, which remains a limitation of the retrieval process.

### 2.2 Inclusion and exclusion criteria

#### 2.2.1 Inclusion criteria

Study Design: Peer-reviewed, randomized controlled trials (RCTs) or quasi-RCTs investigating the acute effects of lower limb BFR training on PAPE and RPE.

Participants: Healthy individuals aged ≤ 45 years, including recreationally active adults, collegiate athletes, or professional athletes (e.g., volleyball, football, track and field) with no history of lower limb musculoskeletal injuries within 6 months of data collection.

Intervention: The experimental group (EG) received lower limb BFR training as the conditioning stimulus for PAPE induction, with clearly documented protocol parameters (e.g., exercise mode, load relative to 1-repetition maximum [1RM], sets, repetitions, BFR pressure [mmHg or % arterial occlusion pressure], and rest intervals).

Comparison: The control group (CG) was assigned to a comparator intervention (e.g., high-load resistance training [≥80% 1RM], low-load resistance training without BFR, or passive rest) with matched exercise mode and volume where applicable.

Outcome Measures: Studies reported quantitative data for at least one primary outcome: (1) PAPE-related explosive performance indicators (e.g., countermovement jump [CMJ] height, in cm or m, mean ± standard deviation [SD]); (2) RPE scores (mean ± SD) for the assessment of subjective fatigue level.

Accessibility: Full-text articles published in the English language.

#### 2.2.2 Exclusion criteria

Study Design: Non-controlled observational studies, systematic reviews, meta-analyses, case reports, conference abstracts, theses, or *in vitro*/animal studies.

Ineligible Interventions: Studies focusing on chronic BFR training adaptations (≥2 weeks) rather than acute PAPE effects; interventions combining BFR with pharmacological agents or other neuromuscular stimulants.

Ineligible Populations: Participants with cardiovascular disease, metabolic disorders, chronic fatigue syndrome, or a history of lower limb surgery (e.g., anterior cruciate ligament reconstruction); pediatric (<18 years) or geriatric (>45 years) populations.

Insufficient Data: Studies lacking extractable quantitative data (mean ± SD, sample size) for PAPE-related performance or RPE scores; reports presenting only qualitative results or aggregate data without group stratification.

Duplicate or Inaccessible Data: Duplicate publications, secondary analyses of the same dataset, or studies with unavailable full texts or unresponsive corresponding authors (where data clarification was requested).

### 2.3 Study selection and data extraction

**Figure 1:**
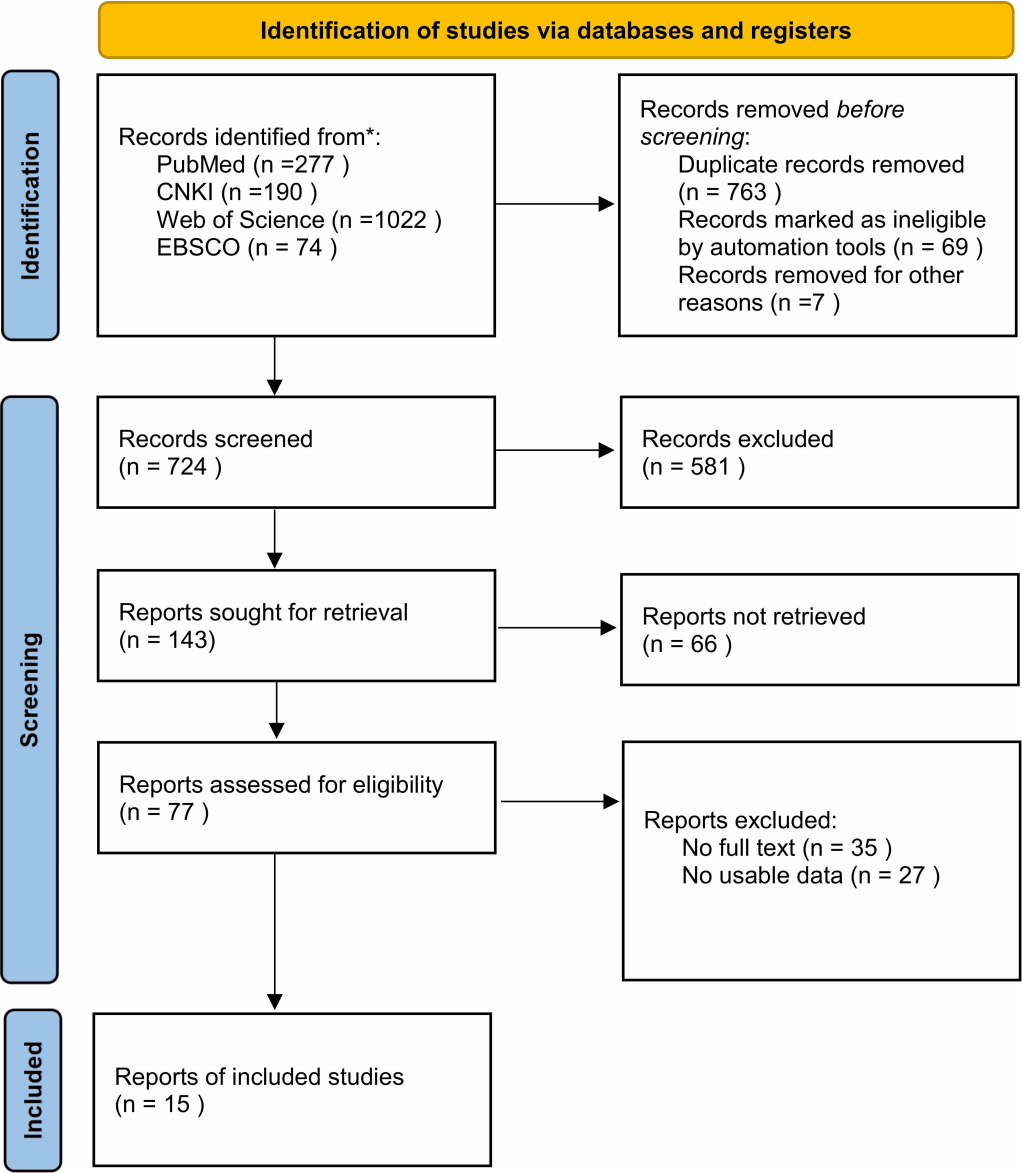
Flow diagram of literature selection.

Two independent reviewers (YL Y and XR M) screened all retrieved records in a two-stage process, in accordance with PRISMA guidelines. First, title and abstract screening was conducted to exclude studies that clearly failed to meet the inclusion criteria; irrelevant records were removed at this stage, and the full texts of potentially eligible studies were obtained for further evaluation. Second, full-text screening was performed to assess compliance with all pre-specified inclusion and exclusion criteria. Any discrepancies between the two reviewers during either screening stage were resolved through consensus discussion with a third independent senior reviewer (WQ C). A PRISMA flow diagram was constructed to report the detailed process of study selection, including the number of records identified, excluded, and ultimately included in the meta-analysis. Ultimately, a total of 15 articles were included in the analysis (**Figure 1**).

### 2.4 Statistical analysis

All statistical analyses were performed using Stata 17.0 and Review Manager. The effect size for continuous outcomes (PAPE-related performance indicators, RPE scores) was calculated as the standardized mean difference (*SMD*) with 95% confidence intervals (*CI)*, using the mean and standard deviation of the experimental and control groups. The random-effects model was applied for all meta-analyses, as this model accounts for between-study heterogeneity and is more conservative for synthesizing heterogeneous data—an expected feature given the variability in BFR protocols and participant characteristics across studies. A fixed-effects model was used only for sensitivity analyses where homogeneity was confirmed.

#### 2.4.1 Heterogeneity assessment

Statistical heterogeneity across included studies was quantified using two tests: the I² statistic and the Cochran’s Q test. The I² statistic was interpreted as follows: *I*² < 25% = low heterogeneity, 25% ≤ *I*² ≤ 50% = moderate heterogeneity, and I² > 75% = high heterogeneity. A p-value < 0.10 for the Cochran’s Q test was considered indicative of statistically significant heterogeneity. Where high heterogeneity (I² > 50%) was detected, subgroup analyses were conducted to explore potential sources of heterogeneity, based on pre-specified moderators: participant training status (recreationally active/athletes), BFR pressure type (absolute [mmHg]/relative [%AOP]), exercise load (%1RM), and exercise mode (squat/leg press/lunge).

#### 2.4.2 Sensitivity analyses

To evaluate the robustness and stability of the meta-analysis results, one-study removal sensitivity analyses were conducted: each included study was sequentially excluded, and the meta-analysis was re-run to determine whether the overall effect size was significantly altered by the exclusion of any single study. This analysis identified potential outlier studies that may have unduly influenced the pooled results. Additional sensitivity analyses were performed by comparing the results of the random-effects and fixed-effects models to assess the impact of statistical model choice on the pooled effect size.

#### 2.4.3 Publication bias assessment

Publication bias (the underreporting of negative or non-significant results) was assessed if the number of included studies for a single outcome was ≥10—an acceptable sample size for valid bias detection. Visual inspection of funnel plots (plot of effect size against standard error) was first used to identify asymmetries indicative of publication bias. Quantitative assessment was then performed using the Egger’s linear regression test (*p* < 0.05 considered indicative of significant publication bias) and the Begg’s rank correlation test (*p* < 0.05 considered indicative of significant publication bias). Where significant publication bias was detected, the trim-and-fill method was applied to adjust the pooled effect size and account for the potential impact of missing studies.

#### 2.4.4 Statistical significance

A two-tailed *p*-value < 0.05 was considered statistically significant for all analyses, including effect size estimates (where the 95% *CI* did not cross 0), heterogeneity tests, subgroup analyses, meta-regression, and publication bias assessments. All pooled effect sizes were interpreted based on Cohen’s guidelines for *SMD: SMD* < 0.2 = trivial effect, 0.2 ≤ *SMD* < 0.5 = small effect, 0.5 ≤ *SMD* < 0.8 = moderate effect, and *SMD* ≥ 0.8 = large effect.

#### 2.4.5 Risk of bias assessment

The original Cochrane Risk of Bias Tool (seven core domains) was used for methodological quality evaluation of all included RCTs. Two independent researchers completed the assessment across seven domains: random sequence generation, allocation concealment, blinding of participants/personnel, blinding of outcome assessment, incomplete outcome data, selective reporting, and other bias. Each domain was rated as low risk, unclear risk, or high risk. Disagreements were resolved via discussion with a third senior researcher.

#### 2.4.6 GRADE evidence certainty assessment

Two independent reviewers conducted GRADE (Grading of Recommendations Assessment, Development and Evaluation) assessment for all primary outcomes and subgroup analyses. Consensus was reached through discussion for inconsistent ratings. The initial evidence grade for RCTs was set as high certainty. We downgraded evidence based on five GRADE domains: risk of bias, inconsistency (heterogeneity), imprecision, indirectness, and publication bias.

Risk of bias: Downgraded for serious risk of bias when more than half of included studies were rated as high/unclear risk in key domains (blinding, allocation concealment).

Inconsistency: Downgraded for substantial heterogeneity (*I*² > 50%).

Imprecision: Downgraded for serious imprecision when total sample size was small (<200 participants) or 95% *CI* crossed the clinical threshold.

Indirectness: Downgraded if study populations, interventions or outcomes deviated from the research question.

Publication bias: No downgrade as funnel plot and Egger’s test showed no significant bias. A supplementary full GRADE evidence profile is provided in the appendix for transparent reference.

## 3 Results

### 3.1 Study characteristics

A pre-piloted, standardized data extraction form was developed and used by the same two independent reviewers to extract relevant data from all included studies, with cross-checking to ensure accuracy. Extracted data were categorized into four core domains:

Study characteristics: First author, publication year, country, study design, sample size (experimental/control groups);
Participant characteristics: Age (mean ± SD), gender, training status (recreationally active/collegiate/professional athlete), sport discipline (where applicable);
Intervention and comparison details: Exercise mode, load (%1RM), sets, repetitions, BFR pressure (mmHg/% arterial occlusion pressure), rest intervals (inter-set/post-conditioning), intervention duration;
Outcome measures: Quantitative data for PAPE-related performance (e.g., CMJ height, mean ± SD) and RPE scores (mean ± SD) at all reported time points, along with the scale used for RPE assessment (e.g., 6–20 Borg scale).

Where key data were missing or incompletely reported in the included studies, the reviewers attempted to contact the corresponding authors via email to request supplementary raw data; studies for which missing data could not be obtained were retained only if extractable data for primary outcomes were sufficient for meta-analysis.

A total of 15 publications were included in this study. All of these publications were randomized controlled trials (RCTs), involving 244 subjects of mixed genders, with ages ranging from 17 to 35 years. The basic characteristics of the included studies are presented in **Table 1**.

**Table 1:** Characteristic of studies included in systematic review and meta-analysis.

Study	Country	Age (years)	N (EG/CG)	Intervention (rest interval)	Plan (BFR intensity)	Outcome extracted
Rivera et al., 2023	USA	21 ± 2	14/14	BFR (Immediate)	4 sets of 30-15-15–15 times 30% 1RM knee extensions(60% AOP)	RPE↑
Wei et al., 2025	China	20.75 ± 2.05	8/8	BFR (4 min)	2 sets × 30 reps, 30% 1RM barbell squats (200 mmHg, 60% AOP)	CMJ↑
Santiago-Pescador et al., 2022	Spain	24.8 ± 7.0	15/15	BFR (6 min)	25 minute single-unit NMES+BFR stimulation protocol (50% AOP)	CMJ↓ RPE↑
Bielitzki et al., 2024	Germany	23.0 ± 2.9	15/15	BFR (4 min)	4 sets (30 - 15 - 15–15 repetitions) of knee extensions at 20%1RM (60%AOP)	RPE↑
Gabrys et al., 2025	Czechia	17 ± 1	20/20	BFR (Immediate)	3 sets × 3 reps on-ice skating (178 ± 13 mmHg, 71 ± 5% AOP)	CMJ↑
Lauver et al., 2017	USA	22.8 ± 2.3	24/24	BFR (Immediate)	4 groups of 30-15-15-15 20%1 RM knee resistance exercises (100% AOP)	MVIC↓
Wernbom et al., 2009	Sweden	25 ± 5	11/11	BFR (3 min)	3 sets of 30%1RM unilateral knee extensions to failure(40% AOP)	MVIC↓ RPE NS
Hornikel et al., 2023	USA	24.8 ± 4.7	13/13	BFR (6 min)	4 sets of 70%1RM squat 4 sets to failure (80% AOP)	CMJ↓ RPE↑
Doma et al., 2020	Australia	22.9 ± 5.0	18/18	BFR (2 min)	3 sets of 8 reps each for single-leg lunges, leg swings, high knees and hip kicks(130mmHg, 50%AOP)	VJ NS RPE↑
Wei et al., 2022 (Hongwen et al., 2022)	China	23.6 ± 1.51	27/27	BFR (15 min)	2 sets of 10 straight leg jumps, followed by 3 sets 5 consecutive obstacle jumps, and then do 5 drop jumps (160mmHg, 40%AOP)	CMJ↑
Sun et al., 2023	China	18.34 ± 1.88	12/12	BFR (8 min)	4 setss of 30-15–15–15 times 30%1RM semi-squat(50%AOP)	CMJ↑ RPE NS
Schwiete et al., 2021	Germany	22.8 ± 1.8	9/10	BFR (6 min)	4 sets of 30-15-15-15 times 20% 1RM leg press(183.3 ± 10mmHg, 60%AOP)	RPE↑
Husmann et al., 2017	Germany	25 ± 4	17/17	BFR (3 min)	4 sets of 30-15-15–15 times 30% 1RM knee extension exercise (208 ± 25 mmHg, 80%AOP)	RPE↑
Cornejo-Daza et al., 2024	Spain	24.5 ± 4.8	20/20	BFR (8 min)	3 sets × 8 reps 60%1RMfull-squat(50%AOP)	CMJ↑
Leszczynski et al., 2024	Australia	18-35	20/20	BFR (Immediate)	3 sets of 5min knee extension exercise(80% AOP)	CMJ↑

NS, no statistical significance; ↑represents a significant increase; ↓ represents a significant decrease; SJ, squat jump; CMJ, counter movement jump; VJ, vertical jump; MVIC, maximum voluntary isometric contraction; AOP, arterial occlusion pressure. Participants in Gabrys et al. (2025) were aged 16–19 years, and most were over 18 years old.

### 3.2 Study quality assessment

**Figure 2:**
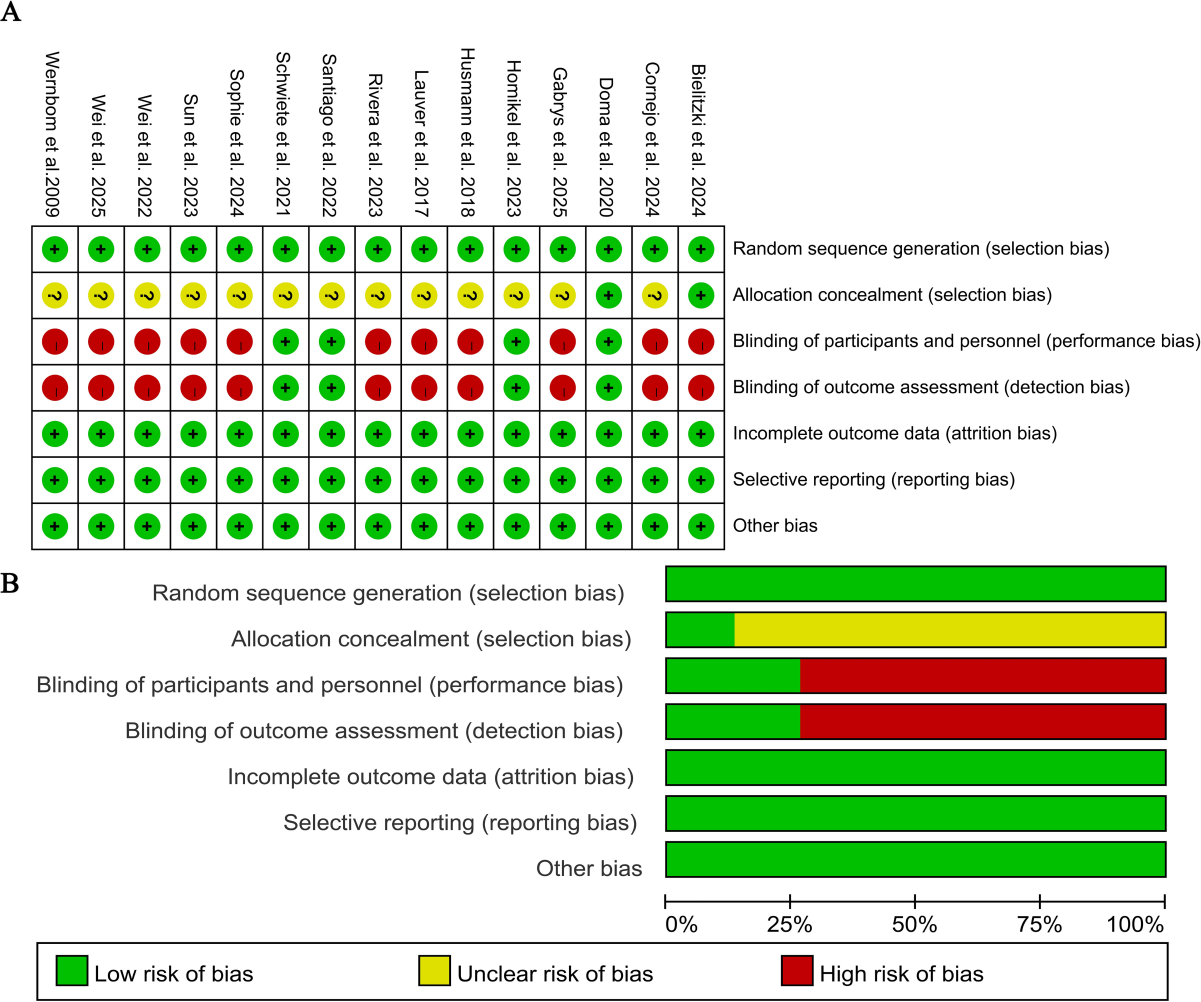
Methodological quality graph and summary of the included studies: **(A)** Risk of bias summary; **(B)** Risk of bias graph;.

The risk of bias in included RCTs was assessed by two reviewers using the original Cochrane Risk of Bias Tool (Higgins et al., 2007), with ratings of low, unclear, or high risk across seven domains: random sequence generation, allocation concealment, blinding of participants/personnel, blinding of outcome assessment, incomplete outcome data, selective reporting, and other bias (**Figures 2A, B**). Disagreements were resolved by a third reviewer. All studies showed low risk for randomization, incomplete data, selective reporting, and other bias. Thirteen had unclear risk for allocation concealment, and 12 had high risk for blinding, as consent was obtained before the trial.

### 3.3 Study quality assessment

We used the GRADE framework to assess the certainty of evidence for primary outcomes. Evidence from RCTs was initially graded as high certainty and downgraded due to risk of bias, heterogeneity, small sample size and indirectness. No significant publication bias was found. The overall evidence certainty for PAPE and RPE was rated low, while all subgroup analyses were judged as very low certainty. Detailed ratings are presented in **Table 2**.

**Table 2:** GRADE certainty of evidence for main outcomes.

Outcome	Studies	Participants	Risk of bias	Heterogeneity	Imprecision	Indirectness	Publication bias	Evidence
PAPE	11	188	Serious	Substantial	Serious	Present	Undetected	Low
RPE	8	121	Serious	Moderate	Serious	Present	Undetected	Low
Subgroup analyses	1–7	14–111	Serious	High	Very serious	Present	Undetected	Very low

### 3.4 Post-activation potentiation

**Figure 3:**
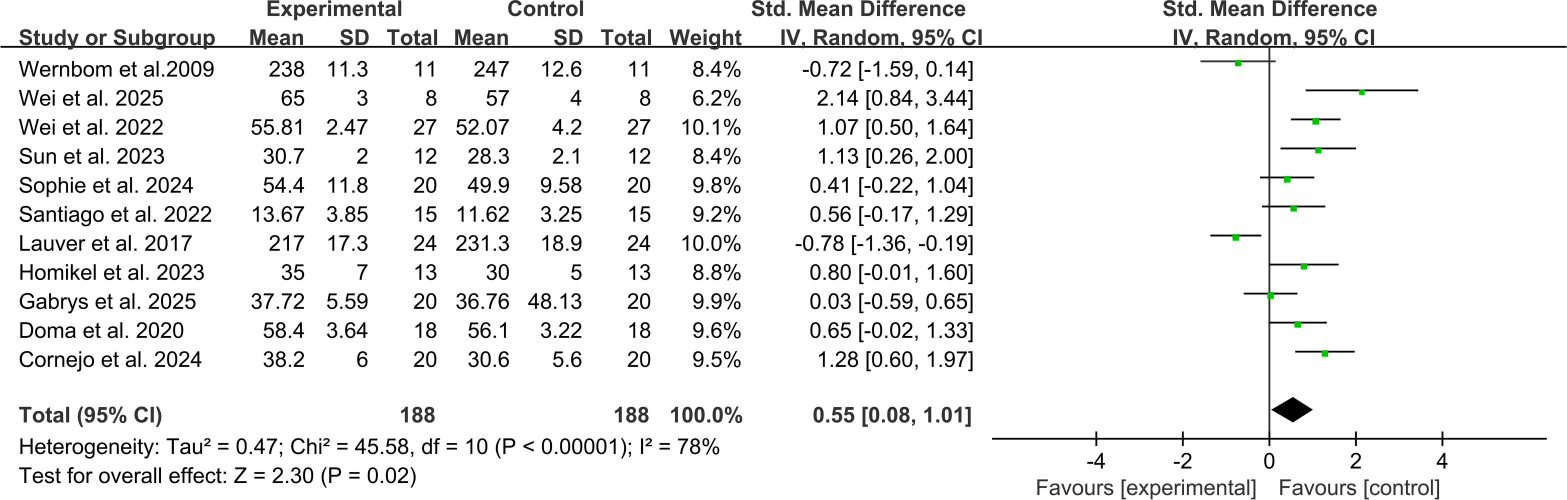
The effect of BFR on PAPE.

A total of 11 studies compared the effects of lower limb BFR training on PAPE, involving 188 subjects in aggregate (**Figure 3**). Heterogeneity testing indicated *I*² = 78% > 50%, and the Q-test yielded *p* < 0.001, suggesting substantial heterogeneity among the included studies. A random-effects model was therefore applied for meta-analysis (**Figure 3**). The results showed a combined effect size *SMD* = 0.55, which was statistically significant (*Z* = 2.30, *p* = 0.02), indicating that lower limb BFR training can significantly improve PAPE-related explosive performance compared with the control condition.

### 3.5 Rating of perceived exertion

**Figure 4:**
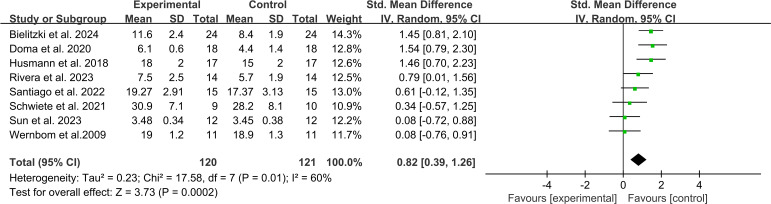
The effect of BFR on RPE.

8 studies reported data on RPE scores to assess subjective fatigue following lower limb BFR training versus control interventions, with a total of 121 subjects (control group) included in this analysis (**Figure 4**). Heterogeneity testing revealed moderate statistical heterogeneity among the studies (*I*² = 60%, *Q* = 17.58, *p* = 0.001). A random-effects model was therefore employed for meta-analysis (**Figure 4**). The pooled effect size was statistically significant [*SMD* = 0.82, 95% *CI*(0.39, 1.26), *Z* = 3.73, *p* < 0.001]. This large effect size indicates that participants in the BFR training group reported significantly higher subjective fatigue levels (higher RPE scores) compared with those in the control group.

### 3.6 Subgroup analysis

Significant heterogeneity (*I*² = 78%) was observed in the pooled effects of BFR training on PAPE. Subgroup analyses identified four primary sources of this variation: exercise mode, rest interval, occlusion pressure, and exercise intensity (**Table 3**).

**Table 3:** Subgroup analysis of BFR training on PAPE and RPE.

Subgroup	Research features	Subgroup standard	Study (sample)	*SMD*	95%*CI*	*P*	*I2* (%)
PAPE	Exercise Mode	Squats	4 (53)	1.20	0.78, 1.63	<0.001	2
		Knee flexion	4 (70)	-0.12	-0.84, 0.59	0.73	76
		Skating	1 (20)	0.03	-0.59, 0.65	0.93	N
		Compound exercise	2 (45)	0.90	0.46, 1.33	<0.001	0
	Rest Interval	Immediate	3 (64)	-0.12	-0.81, 0.57	0.74	74
		2 ~4 min	3 (37)	0.62	-0.77, 2.07	0.38	86
		> 4 min	5 (87)	0.98	0.67, 1.30	<0.001	0
	Compressive Strength	40 ~ 60% AOP	7 (111)	0.83	0.29, 1.36	0.002	70
		> 60% AOP	4 (77)	0.08	-0.57, 0.74	0.80	75
	Exercise Intensity	Self weight	5 (81)	0.58	0.10, 1.07	0.02	54
		< 40% 1RM	3 (63)	0.46	-0.85, 1.76	0.49	91
		40 ~70%% 1RM	1 (20)	1.28	0.60, 197	<0.001	N
		Exhaustive exercise	2 (24)	0.05	-1.44, 1.53	0.95	84
RPE	Exercise Mode	Squats	3 (35)	0.55	-0.16, 1.26	0.13	52
		Knee flexion	5 (81)	0.91	0.40, 1.41	<0.001	57
		Compound exercise	1 (18)	1.54	0.79, 2.30	<0.001	N
	Rest Interval	Immediate	1 (14)	0.79	0.001, 1.56	0.05	N
		2 ~ 4 min	4 (70)	1.16	0.52, 1.80	<0.001	66
		> 4 min	4 (50)	0.57	0.08, 1.05	0.02	28
	Exercise Intensity	Self weight	2 (33)	1.07	0.16, 1.99	0.02	67
		< 40% 1RM	5 (77)	0.86	0.30, 1.42	0.003	62
		Exhaustive exercise	2 (24)	0.66	-0.49, 1.82	0.26	73
	Compressive Strength	40 ~ 60% AOP	7 (104)	0.73	0.27, 1.19	0.002	60
		> 60% AOP	2 (30)	1.37	0.80, 1.94	<0.001	0

N, no applicable.

Exercise Mode: Squats (*SMD* = 1.20, 95% *CI*: 0.78–1.63, *P <* 0.001) and compound exercise (*SMD* = 0.90, 95% *CI*: 0.46–1.33, *P <* 0.001) yielded large, statistically significant effects, with low heterogeneity (I² = 2% and 0%, respectively).

In contrast, knee flexion (*SMD* = -0.12, *p* = 0.73) and skating (*SMD* = 0.03, *p* = 0.93) showed no significant effect, with high heterogeneity (*I*² = 76%) observed in the knee flexion subgroup.

Rest Interval: Longer rest intervals (> 4 min) produced a significant positive effect (*SMD* = 0.98, 95% *CI*: 0.67–1.30, *P < 0.001*) with no heterogeneity (*I*² = 0%). Moderate rest intervals (2–4 min) showed a moderate effect (*SMD* = 0.62, *p* = 0.38) with high heterogeneity (*I*² = 86%), while immediate rest intervals showed no significant effect (*SMD* = -0.12, *p* = 0.74, *I²* = 74%).

occlusion pressure: Moderate occlusion pressure (40–60% AOP) resulted in a significant positive effect (*SMD* = 0.83, 95% *CI*: 0.29–1.36, *p* = 0.002) with high heterogeneity (*I*² = 70%). Higher occlusion pressure (> 60% AOP) showed no significant effect (SMD = 0.08, p = 0.80) with high heterogeneity (I² = 75%).

Exercise Intensity: Moderate intensity (40–70% 1RM) produced a large, significant effect (SMD = 1.28, 95% CI: 0.60–1.97, p < 0.001) with no heterogeneity (I² = 0%). This subgroup was derived from only a single study (n = 20), and the observed effect cannot be generalized as a valid subgroup-level conclusion. Lower intensity (< 40% 1RM) and exhaustive exercise showed no significant effect, with high heterogeneity (*I*² = 91% and 84%, respectively). Self-weight exercise showed a moderate effect (*SMD* = 0.58, *p* = 0.02) with moderate heterogeneity (*I*² = 54%).

Outcome Extracted (PAPE): CMJ testing showed a significant positive effect (*SMD* = 0.83, 95% *CI*: 0.44–1.21, *P < 0.001*) with moderate heterogeneity (*I*² = 54%). MVIC testing showed a significant negative effect (*SMD* = -0.76, 95% *CI*: -1.25 to -0.27, *p* = 0.002) with no heterogeneity (*I*² = 0%). VJ testing showed no significant effect (*SMD* = 0.65, *p* = 0.06) with no heterogeneity (*I*² = 0%).

RPE Outcomes: Significant heterogeneity (*I*² = 60%) was observed in the pooled effects of BFR training on RPE. Subgroup analyses revealed the following key findings:

Exercise Mode: Knee flexion (*SMD* = 0.91, 95% *CI*: 0.40–1.41, *p* < 0.001) and compound exercise (*SMD* = 1.54, 95% *CI*: 0.79–2.30, *P < 0.001*) produced large, significant effects with moderate heterogeneity (I² = 57% and 0%, respectively). Squats showed a moderate effect (*SMD* = 0.55, *p* = 0.13) with low heterogeneity (*I*² = 52%).

Rest Interval: Immediate rest intervals (*SMD* = 0.79, 95% *CI*: 0.001–1.56, *p* = 0.05) and 2–4 min rest intervals (*SMD* = 1.16, 95% *CI:* 0.52–1.80, *p* < 0.001) showed significant effects, with high heterogeneity (*I*² = 66%) in the 2–4 min subgroup. Longer rest intervals (> 4 min) showed a moderate effect (*SMD* = 0.57, 95% *CI*: 0.08–1.05, *p* = 0.02) with low heterogeneity (*I*² = 28%).

Exercise Intensity: Self-weight (*SMD* = 1.07, 95% *CI:* 0.16–1.99, *p* = 0.02) and < 40% 1RM (*SMD* = 0.86, 95% *CI*: 0.30–1.42, *p* = 0.003) showed significant effects with moderate heterogeneity (*I*² = 67% and 62%, respectively). Exhaustive exercise showed no significant effect (*SMD* = 0.66, *p* = 0.26) with high heterogeneity (*I*² = 73%).

occlusion pressure: Both 40–60% AOP (*SMD* = 0.73, 95% *CI*: 0.27–1.19, *p* = 0.002) and > 60% AOP (*SMD* = 1.37, 95% *CI*: 0.80–1.94, *P < 0.001*) produced significant effects, with no heterogeneity (*I*² = 0%) in the > 60% AOP subgroup.

### 3.7 Sensitivity analysis

Sensitivity analysis was conducted using the leave-one-out method to evaluate the robustness of the results.

As shown in **Table 4**, the pooled effect size of BFR training on PAPE was *SMD* = 0.55, 95% *CI* (0.08, 1.01), *p* = 0.02. After sequentially removing individual studies, the pooled *SMD* ranged from 0.44 to 0.66, and the heterogeneity index *I*² varied between 76% and 86%. All results remained statistically significant (*p* < 0.05).

**Table 4:** Combined effects of jumping ability after excluding individual studies.

Subgroup	Study	*SMD*	95%*CI*	*P*	*I2* (%)
PAPE	Bielitzki et al., 2024	0.61	0.10, 1.11	0.02	79
	Cornejo et al., 2024	0.68	0.29, 1.08	0.001	65
	Doma et al., 2020	0.54	0.02, 1.05	0.04	80
	Gabrys et al., 2025	0.57	0.04, 1.09	0.03	80
	Homikel et al., 2023	0.44	-0.001, 0.90	0.05	77
	Husmann et al., 2018	0.66	0.20, 1.12	0.005	76
	Lauver et al., 2017	0.47	-0.001, 0.95	0.06	77
	Rivera et al., 2023	0.52	0.02, 1.03	0.04	80
	Santiago et al., 2022	0.55	0.04, 1.06	0.04	80
	Schwiete et al., 2021	0.49	-0.001, 0.99	0.05	78
	Sophie et al., 2024	0.49	0.00, 0.98	0.05	79
	Overall	0.55	0.08, 1.01	<0.02	78
RPE	Bielitzki et al., 2024	0.78	0.37,1.20	<0.001	53
	Doma et al., 2020	0.78	0.38,1.19	<0.001	54
	Homikel et al., 2023	0.87	0.48, 1.26	<0.001	56
	Husmann et al., 2018	0.79	0.38,1.21	<0.001	56
	Rivera et al., 2023	0.88	0.43, 1.32	<0.001	62
	Santiago et al., 2022	0.90	0.46, 1.34	<0.001	60
	Schwiete et al., 2021	0.93	0.51, 1.34	<0.001	58
	Sun et al., 2023	0.97	0.59, 1.36	<0.001	49
	Wernbom et al.2009	0.97	0.58, 1.35	<0.001	50
	Overall	0.82	0.39,1.26	<0.001	60

For RPE, the overall pooled effect size was *SMD* = 0.82, 95% *CI* (0.39, 1.26), *p* < 0.001. After sequentially removing each study, the pooled *SMD* ranged from 0.78 to 0.97, with *I*² values between 49% and 62%. All results remained statistically significant (*p* < 0.002). No single study was found to unduly influence the overall effect sizes, indicating that the findings of this meta-analysis are relatively stable and robust.

### 3.8 Publication bias

**Figure 5:**
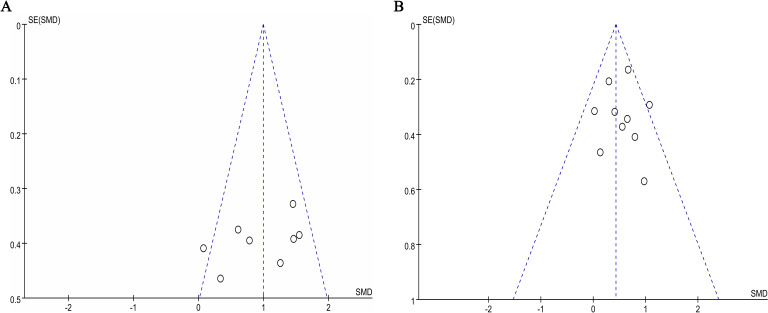
Funnel plot: **(A)** PAPE; **(B)** RPE.

This study constructed funnel plots for each subgroup to assess potential publication bias. As shown in **Figure 5**, the funnel plots exhibited an approximately symmetrical shape. Egger’s test was further conducted on these funnel plots, and the results showed that the p-values for all subgroups were greater than 0.05, indicating no significant publication bias among the included studies.

## 4 Discussion

### 4.1 Impact of BFR training on PAPE

This meta-analysis systematically synthesized data from 11 studies involving 188 participants to explore the effect of lower limb BFR training on PAPE. The pooled results revealed a statistically significant moderate effect (*SMD* = 0.55, 95% *CI*: 0.08–1.01, *p* = 0.02), confirming that lower limb BFR training can effectively enhance PAPE-related explosive performance compared with control conditions. This finding is consistent with speculative physiological hypotheses from previous literature. By partially restricting limb blood flow, BFR may lead to metabolite accumulation (e.g., hydrogen ions, inorganic phosphates) during muscle contraction. Previous studies hypothesized that Such metabolic changes could lower intramuscular pH, which may potentially modify the conformation of contractile proteins and improve calcium sensitivity (this mechanism was not measured in included trials), and may thereby enhance muscle contraction efficiency and potentially induce PAPE (Wang et al., 2023; Lubiak et al., 2025). Additionally, existing literature proposes that BFR-induced metabolic stress activates intracellular signaling pathways which may promote muscle protein synthesis, potentially facilitates muscle hypertrophy, and may further strengthens force-generating capacity, which is speculated to contribute to PAPE induction (Loenneke et al., 2012). Consistent with this, prior small-sample studies (e.g., Wei et al., 2025; Sun et al., 2023) have reported that BFR training can acutely enhance neuromuscular potentiation, verifying the reproducibility of the promoting effect of BFR on PAPE.

Two included studies reported contradictory negative outcomes, with Wernbom et al. (2009) and Lauver et al. (2017) yielding negative effect sizes (SMD = −0.72 and SMD = −0.78, respectively), indicating impaired neuromuscular potentiation following BFR conditioning, which seemingly conflicts with the overall positive pooled PAPE effect in this meta-analysis. Specifically, Wernbom et al. (2009) implemented a training protocol performed to failure, which inevitably induced substantial accumulated fatigue that completely offset the BFR-induced potentiation response and ultimately suppressed PAPE performance. Second, Lauver et al. (2017) applied relatively higher BFR pressure combined with a short post-conditioning rest interval, which excessively amplified BFR-triggered metabolic stress. Additionally, the untrained and recreationally active participants recruited in these two studies possessed low neuromuscular tolerance to intense BFR-induced metabolic perturbation, making them more vulnerable to fatigue accumulation and performance decline. Despite these individual negative findings, the overall pooled result remained significantly positive, demonstrating that such fatigue-dominated inhibitory effects are study-specific and isolated cases rather than universal responses. These discrepancies further highlight that the PAPE-promoting effect of lower limb BFR training is highly protocol-dependent, and rational manipulation of training intensity, BFR pressure, and rest duration is essential to balance potentiation and fatigue and achieve optimal training outcomes.

Notably, high heterogeneity was observed among the included studies (*I*² = 78%, *p* < 0.001), which may stem from differences in BFR protocol parameters, participant characteristics, and PAPE measurement methods. To clarify the sources of heterogeneity, subgroup analyses were conducted based on exercise mode, rest interval, occlusion pressure, exercise intensity, and outcome measures, yielding targeted insights for practical application.

#### 4.1.1 Exercise mode

Subgroup analysis showed that squats (*SMD* = 1.20, *P < 0.001*, *I*² = 2%) and compound exercises (*SMD* = 0.90, *P < 0.001*, *I*² = 0%) produced large and statistically significant PAPE effects with low heterogeneity. It should be emphasized that the compound exercise subgroup only included 2 studies, so this conclusion has limited generalizability. This outcome is speculatively explained by existing physiological theories: squats and compound exercises engage multi-joint, multi-muscle group activation (e.g., quadriceps femoris, gluteus maximus, hamstrings), which maximizes metabolic stress under BFR conditions. The synchronous contraction of multiple muscle groups amplifies the accumulation of metabolites, and prior literature hypothesizes that this may intensify the stimulation of type II muscle fibers—key for explosive performance (no direct evidence from included studies) (Drust et al., 2007). In contrast, knee flexion (*SMD* = -0.12, *p* = 0.73, *I*² = 76%) and skating (*SMD* = 0.03, *p* = 0.93) showed no significant PAPE effects. The skating subgroup was based on a single study, and this result cannot be widely applied. Knee flexion primarily targets isolated muscle groups (e.g., hamstrings), resulting in limited metabolic stress accumulation and insufficient stimulation of neuromuscular potentiation (Xie et al., 2025). For skating, as a sport-specific on-ice movement, environmental factors (e.g., ice surface friction) and the complexity of coordinated limb movements may dissipate the mechanical and metabolic stimuli induced by BFR, thereby offsetting the PAPE-promoting effect (Lin et al., 2025). These results suggest that BFR training should prioritize multi-joint compound movements such as squats to optimize PAPE outcomes, as they better align with the physiological mechanisms of BFR-induced potentiation proposed in previous research.c

#### 4.1.2 Rest interval

Longer rest intervals (> 4 min) yielded the most significant PAPE effect (*SMD* = 0.98, *P < 0.001*, *I*² = 0%), while moderate (2–4 min) and immediate rest intervals showed no significant effects (*SMD* = 0.62, *p* = 0.38; *SMD* = -0.12, *p* = 0.74). This pattern reflects the physiological balance between PAPE and fatigue: immediate or short rest intervals result in unresolved metabolic fatigue (e.g., lactic acid accumulation, muscle fiber microdamage), which masks the potentiation effects induced by BFR (McGowan et al., 2015). Longer rest intervals (> 4 min) are hypothesized to allow for partial clearance of fatigue-related metabolites through restored blood flow, while potentially retaining the residual potentiation related to myosin light chain phosphorylation and increased neural drive (these mechanisms were not tested in included trials) (Zhou et al., 2026). For example, Wei et al. (2022) found that a 15-min rest interval after BFR training significantly improved CMJ height, which supports the superiority of longer rest intervals in PAPE induction. The absence of heterogeneity in the > 4 min subgroup further confirms that this rest interval window is robust across different study populations and protocols.

#### 4.1.3 Occlusion pressure

Moderate occlusion pressure (40–60% AOP) produced a significant PAPE effect (*SMD* = 0.83, *p* = 0.002), while higher occlusion pressure (> 60% AOP) showed no significant effect (*SMD* = 0.08, *p* = 0.80). Moderate AOP (40–60%) achieves an optimal balance between blood flow restriction and tissue perfusion: it sufficiently reduces venous return to accumulate metabolic stress, triggering PAPE, without causing severe arterial occlusion that would limit oxygen and nutrient supply to muscles (Schoenfeld et al., 2017). In contrast, occlusion pressure exceeding 60% AOP may lead to near-complete arterial occlusion, resulting in tissue hypoxia, increased muscle damage, and excessive fatigue—all of which inhibit neuromuscular potentiation (Li et al., 2025). This finding aligns with BFR training consensus guidelines, which emphasize that individualized AOP titration (based on limb circumference and vascular compliance) is critical to avoid adverse effects and maximize training benefits (Baker et al., 2020). The high heterogeneity in the > 60% AOP subgroup is speculated to relate to inter-individual differences in vascular reactivity (no direct evidence from included studies) and tolerance to extreme occlusion.

#### 4.1.4 Exercise intensity

Moderate exercise intensity (40–70% 1RM) produced the largest PAPE effect (*SMD* = 1.28, *p* = 0.001), but this result was derived from only one single study and lacks generalizability,while lower intensity (< 40% 1RM) and exhaustive exercise showed no significant effects. Moderate intensity (40–70% 1RM) combined with BFR creates a synergistic effect: the mechanical load provides sufficient stimulation to activate muscle fibers, while BFR amplifies metabolic stress—together as hypothesized in prior studies optimizing the conditions for PAPE induction (de Lemos Muller et al., 2024). Lower intensity (< 40% 1RM) fails to generate adequate mechanical tension or metabolic stress, resulting in weak potentiation, while exhaustive exercise induces overwhelming fatigue that offsets the beneficial effects of BFR (Fry et al., 1997). Self-weight exercise (*SMD* = 0.58, *p* = 0.02) also showed a moderate PAPE effect, indicating that BFR can enhance potentiation even without external loads. This is particularly valuable for populations with limited load-bearing capacity (e.g., rehabilitation patients), as self-weight BFR training reduces injury risk while maintaining neuromuscular function through metabolic stress-induced potentiation (Trybulski et al., 2026).

#### 4.1.5 Outcome measures

CMJ (*SMD* = 0.83, *P <* 0.001) emerged as the most sensitive outcome measure for PAPE assessment, as it reflects integrated lower limb explosive power and dynamically engages the neuromuscular pathways targeted by BFR training (Brodt et al., 2008). MVIC showed a significant negative effect (*SMD* = -0.76, *p* = 0.002), which may be due to the isometric nature of MVIC: BFR-induced metabolic fatigue has a more pronounced inhibitory effect on isometric contractions, where muscle fibers are held in a fixed position without the mechanical advantage of dynamic length changes (Burnstein et al., 2011). VJ showed no significant effect (*SMD* = 0.65, *p* = 0.06), possibly due to variations in movement standardization (e.g., arm swing usage, jump initiation technique) across studies, which introduced measurement variability. These results highlight that CMJ is the preferred outcome measure for evaluating BFR-induced PAPE, as it more reliably captures the dynamic potentiation effects relevant to sport-specific performance.

### 4.2 Impact of BFR training on RPE

This meta-analysis analyzed data from 8 studies involving 121 participants to explore the effect of lower limb BFR training on RPE. The pooled results showed a statistically significant large effect (SMD = 0.82, 95% CI: 0.39–1.26, p < 0.001), indicating that BFR training significantly increases subjective fatigue perception compared with control conditions. The underlying mechanisms are multi-faceted and are all speculative based on published literature, not direct measurements from included trials:1) Metabolite accumulation: BFR restricts venous return, leading to the accumulation of hydrogen ions, lactic acid, and other metabolites in muscles, which stimulates type III/IV afferent nerve fibers—specialized for detecting metabolic stress—and transmits fatigue signals to the central nervous system (Liu et al., 2023); 2) Enhanced neuromuscular activation: BFR increases the discharge frequency of muscle spindles and Golgi tendon organs, amplifying neural feedback to the brain and elevating perceived load (Ahmed et al., 2024); 3) Attention focus: Individuals undergoing BFR training are more attentive to physical sensations (e.g., limb pressure, muscle tightness), which amplifies the perception of fatigue (Englund et al., 2022). Collectively, these factors are hypothesized to explain why BFR training induces higher subjective fatigue despite lower external loads compared to conventional resistance training.

Moderate heterogeneity was observed among the included studies (*I*² = 60%, *p* = 0.001), which may be attributed to differences in exercise mode, rest interval, exercise intensity, and occlusion pressure. Subgroup analyses were conducted to further explore these factors.

#### 4.2.1 Exercise mode

Knee flexion (*SMD* = 0.91, *p* < 0.001, *I*² = 57%) and compound exercises (*SMD* = 1.54, *P < 0.001*) produced large and significant RPE effects. The compound exercise subgroup for RPE contained only one study, so this finding has limited reference value. Knee flexion primarily targets the thigh muscles (quadriceps femoris, hamstrings), and BFR-induced blood flow restriction leads to rapid metabolite accumulation in these isolated muscle groups—concentrating the stimulation of fatigue receptors and afferent nerve fibers (Noh et al., 2020). Compound exercises involve multi-muscle group activation, resulting in greater overall metabolic stress and a more widespread perception of fatigue (de Queiros et al., 2021). Squats showed a moderate effect (*SMD* = 0.55, *p* = 0.13, *I*² = 52%), which may be due to the involvement of larger muscle groups (e.g., gluteus maximus) that distribute metabolic stress across a broader tissue area, reducing localized fatigue perception compared to knee flexion. Additionally, squats engage core stabilizers, which may divert attention from limb-specific fatigue signals, further mitigating RPE (Cerqueira et al., 2022).

#### 4.2.2 Rest interval

Immediate rest intervals (*SMD* = 0.79, *p* = 0.05) and moderate rest intervals (2–4 min, *SMD* = 1.16, *p* < 0.001) showed significant RPE effects, while longer rest intervals (> 4 min) produced a smaller moderate effect (*SMD* = 0.57, *p* = 0.02). Immediate and short rest intervals result in minimal metabolite clearance, maintaining high levels of metabolic stress and sustained activation of fatigue-sensitive nerve fibers (Pearson et al., 2015). Longer rest intervals allow for partial metabolite clearance via restored blood flow, reducing the intensity of fatigue signals and lowering subjective perception (Silva et al., 2018). This finding is consistent with the PAPE subgroup analysis, highlighting the inherent trade-off between potentiation and fatigue: longer rest intervals optimize PAPE by resolving fatigue, while shorter rest intervals preserve metabolic stress but increase RPE. For practical application, this suggests that the choice of rest interval should be tailored to training goals. For clinical rehabilitation, longer rest intervals are preferred for vulnerable populations (post-surgical and elderly patients) to avoid excessive RPE and ensure training safety, while short rest intervals are only indicated for low-risk patients in late rehabilitation aiming to improve metabolic adaptation and muscular endurance.

#### 4.2.3 Exercise intensity

Self-weight exercise (*SMD* = 1.07, *p* = 0.02, *I*² = 67%) and low intensity (< 40% 1RM, *SMD* = 0.86, *p* = 0.003, *I*² = 62%) produced significant RPE effects. Under BFR conditions, self-weight and low-intensity exercises require higher repetitions (e.g., 30–45 repetitions per set) to achieve sufficient metabolic stimulation, leading to prolonged muscle activation and increased fatigue perception (Centner et al., 2019). The cumulative duration of muscle contraction amplifies metabolite accumulation and neural feedback, even in the absence of high external loads. Exhaustive exercise showed no significant effect (*SMD* = 0.66, *p* = 0.26, *I*² = 73%), which may be due to the ceiling effect of RPE: individuals reach maximal fatigue perception during exhaustive exercise regardless of BFR, masking group differences (Martín-Hernández et al., 2017). Additionally, exhaustive exercise may induce central fatigue, which overrides the peripheral fatigue signals specific to BFR, further reducing the detectability of group differences.

#### 4.2.4 Occlusion pressure

Both moderate (40–60% AOP, *SMD* = 0.73, *p* = 0.002) and high (> 60% AOP, *SMD* = 1.37, *P < 0.001*) occlusion pressures produced significant RPE effects, with high AOP showing a larger effect size. Higher occlusion pressure (> 60% AOP) leads to more severe blood flow restriction, reducing muscle oxygen supply and accelerating anaerobic metabolism—greatly increasing lactic acid accumulation and the stimulation of metabolic pain receptors (Zeng et al., 2025). Additionally, high AOP may cause physical discomfort (e.g., limb numbness, vascular distension), which is interpreted by the central nervous system as a fatigue signal, further elevating RPE (Serrano-Ramón et al., 2025). This finding contrasts with the PAPE subgroup analysis, where high AOP failed to enhance potentiation but significantly increased fatigue—indicating that occlusion pressure exceeding 60% AOP is impractical for balancing performance gains and tolerability. The lack of heterogeneity in the > 60% AOP subgroup suggests that the adverse fatigue effects of high occlusion are consistent across studies, reinforcing the need to avoid excessive AOP in BFR training protocols.

### 4.3 Limitations and future directions

This meta-analysis has several limitations. First, the number of included studies is relatively small (15 studies), and the sample size of individual studies is limited, which may affect the statistical power and generalizability of the results. Second, high heterogeneity was observed in some subgroups, which may be due to unmeasured moderators (e.g., participant gender, training experience, BFR device type). Most importantly, we failed to perform stratified analysis based on participants’ training status (recreationally active individuals, amateur athletes, professional athletes). Training status is a core factor affecting PAPE response, and the inability to stratify and explore this moderator is a major limitation of this meta-analysis, which is one of the key sources of high between-study heterogeneity. Third, the included studies lacked standardized BFR protocols (e.g., pressure setting methods, exercise volume), making it difficult to determine the optimal protocol for PAPE induction. Fourth, the search strategy has inherent limitations: combined RCT and RPE keywords may exclude eligible literature, and no supplementary sensitivity analysis was conducted for retrieval results. Fifth, multiple subgroups were based on only 1–2 studies, and these small-sample findings have poor external validity. Sixth, publication bias cannot be completely excluded, although funnel plots and Egger’s test showed no significant bias.

Future research should address these limitations by: (1) Conducting large-sample, multi-center RCTs to verify the effect of BFR training on PAPE and RPE; (2) Standardizing BFR protocols (e.g., individualized AOP titration, unified exercise mode and volume) to reduce heterogeneity; (3) Strictly classifying participants according to training status and performing stratified analysis to clarify the differentiated effects of BFR training on different populations; (4) Optimizing literature search strategies and conducting sensitivity analysis for retrieval results to reduce omission bias; (5) Focusing on mechanistic research to directly detect physiological indicators (e.g., calcium sensitivity, myosin light chain phosphorylation) and verify the hypotheses proposed in this study; (6) Validating small-sample subgroup conclusions with more independent trials.

## 5 Conclusion

Lower limb blood flow restriction (BFR) training can effectively induce PAPE, with moderate exercise intensity of 40–70% 1RM, occlusion pressure of 40–60% arterial occlusion pressure (AOP), rest intervals of more than 4 minutes, and multi-joint movements such as squats being more conducive to the enhancement of PAPE effects. It should be noted that some optimal parameters are derived from single or a small number of studies and need further verification. Meanwhile, BFR training significantly elevates subjective fatigue levels as assessed by the Rating of Perceived Exertion (RPE), and the fatigue perception is more prominent when BFR training is combined with knee flexion or compound exercises, accompanied by rest intervals of 4 minutes or less, low exercise intensity of less than 40% 1RM, or occlusion pressure of more than 60% AOP. Several RPE subgroup results are based on limited studies and cannot be widely promoted.

## Data Availability

The datasets presented in this study can be found in online repositories. The names of the repository/repositories and accession number(s) can be found in the article/supplementary material.
